# Experimental Study on Multi-Dimensional Visualization Simulation of Gas and Gel Foam Flooding in Fractured-Vuggy Reservoirs

**DOI:** 10.3390/gels9090722

**Published:** 2023-09-06

**Authors:** Yuchen Wen, Jirui Hou

**Affiliations:** Key Laboratory of Petroleum Engineering, Research Institute of Unconventional Petroleum Science and Technology, China University of Petroleum (Beijing), Beijing 102249, China

**Keywords:** fractured-vuggy reservoirs, foam flooding, gas flooding, visualization simulation, EOR

## Abstract

Gas flooding and foam flooding are potential technologies for tertiary oil recovery in fractured-vuggy reservoirs. The development and mechanism research of fractured-vuggy reservoirs is difficult due to the complex structures and the strong heterogeneity of fractured-vuggy reservoirs. Visualization simulation is one of the effective methods to study the flow behavior of fluid in fractured-vuggy reservoirs. In this study, an upscaling method of visualization simulation from one dimension (1D) to three dimensions (3D) was established, and the physical models of fractured-vuggy reservoirs were designed and fabricated. Water flooding, gas flooding, and gel foam flooding were carried out in the models. The experimental results showed that gas flooding has a single flow channel and water flooding has multiple flow channels in fractures and vugs. Gel foam with an excellent capability of mobility control and a high microscopic displacement efficiency swept in all directions at a uniform velocity. The EOR mechanisms of gel foam in fractured-vuggy reservoirs were mainly as follows: reducing interfacial tension, increasing mobility ratio, selectively plugging high permeability channels, and discontinuous flow. In the displacement process of fractured-vuggy reservoirs, water should be injected from the well at the bottom of the reservoir, and gas should be injected from the well located in the vug at the high part of the reservoir. Gel foam with strong stability and high viscosity should be selected and injected in most kinds of injection wells in fractured-vuggy reservoirs. This study provides a complete method of visualization simulation for the study of flow behavior in fractured-vuggy reservoirs and provides theoretical support for the application of gas flooding and gel foam flooding in fractured-vuggy reservoirs.

## 1. Introduction

Carbonate reservoirs play an important role in the world’s oil and gas resources, and their oil and gas reserves account for 70% of the world’s total oil and gas reserves [1]. Carbonate reservoirs have the characteristics of large-scale and high production. Among them, fractured-vuggy reservoirs account for more than 30% of carbonate reservoirs [2]. Carbonate sedimentary rocks in China are mainly distributed in the Tarim Basin, the Sichuan Basin, the Ordos Basin, and North China, among which the most typical fractured-vuggy reservoir is the Ordovician carbonate reservoir in the Tahe Oilfield [3]. The main structures of the reservoir are fractures and secondary pores, indicating that the reservoir space of oil is mainly fractures and vugs. The matrix permeability of the carbonate reservoir is extremely low (less than 1 mD). The flow ability of fluid in the matrix is extremely poor, and fractures are the main channels [4,5].

After multiple tectonic processes, many fractures and vugs are crisscross developed in fracture-vuggy carbonate reservoirs. The complexity of the connection between fractures and vugs results in the strong heterogeneity of fracture-vuggy carbonate reservoirs [6]. There is a big difference in the size of the fractured-vuggy structures and the regularity of the connection mode. The strong heterogeneity in the spatial structure distribution of fractured-vuggy carbonate reservoirs resulted in great difficulty in the development and low oil recovery of fracture-cave carbonate reservoirs [7]. The Tahe Oilfield was mainly developed by natural energy in the early stage of development, and the average oil recovery ratio was 12.4% [8]. Subsequent water flooding was carried out in different ways, but laboratory experiments and field applications have proved that the development effect of water flooding in fractured-vuggy reservoirs was extremely limited [9,10]. By 2009, the oil recovery ratio in the stage of water flooding increased by 2.45% [8]. By drawing lessons from the successful cases of gas flooding in the United States and other countries, field tests of gas flooding were carried out in the Tahe Oilfield in 2017 [11]. Gas replaced the residual oil in the high part of fractured-vuggy reservoirs by gravity differentiation. Due to the special structure of the fractured-vuggy reservoir, gas channeling easily occurs in reservoirs [12,13]. By 2019, the oil recovery ratio in the stage of gas flooding increased by 2.16%, which is not satisfactory [8]. Foam and nanoparticles are potential technologies for enhanced oil recovery in fractured-vuggy reservoirs [14,15,16]. Laboratory experiments showed that foam could not only expand the swept volume but also improve the displacement efficiency, thus significantly improving the oil recovery of fractured-vuggy reservoirs [17]. In recent years, some progress has been made in the application of foam flooding in fractured-vuggy reservoirs. Foam has been proven to be beneficial in improving the gas flooding effect in fractured-vuggy reservoirs [18].

With the continuous development and understanding of carbonate reservoirs, the theory of the “fractured-vuggy unit” was put forward to reasonably classify the reservoir space of the fractured-vuggy reservoir. The basic unit is a separate fractured-vuggy unit, and the fractured-vuggy reservoir system is composed of multiple basic units through different combinations [7,19]. This classification method not only provided a geological basis for the development of fractured-vuggy carbonate reservoirs but also provided a theoretical basis for experimental research and the establishment of physical models.

On this basis, a large number of studies were carried out by researchers on numerical simulations [20,21,22,23], physical simulations [24,25] and other methods. However, because of the unique geological characteristics and complex reservoir environment of the fractured-vuggy reservoir, it is very difficult to study the flow law of fluids and the development effect of reservoirs in the fractured-vuggy reservoir. Visualization simulation is still one of the most intuitive and effective methods to study the law of fluid flow in fractured-vuggy reservoirs.

Table 1 was a partial statistic on visualization simulation of fractured-vuggy reservoirs. Y. Wen. used a one-dimensional (1D) visual model to reveal the microscopic flow characteristics and displacement effect of foam in fractures [15]. Yang used a 1D visual model to reveal the influence of the shape of fracture-vuggy structure on fluid flow and residual oil distribution [26]. A two-dimensional (2D) model was established by Hou [27], and the EOR effect of water flooding and nitrogen gas flooding in fractured-vuggy structures was verified. On this basis, Qu designed a similar 2D visual model to study foam flooding and revealed that the main EOR mechanism of foam flooding was the reduction of oil–water interfacial tension and gravity differentiation [28]. Wang verified the displacement law and the EOR effect of gas flooding in fractured-vuggy reservoirs by using a 2D visual model [29]. Liang and Hou also established a new kind of 2D visual model and clarified the flow characteristics and phase behavior of nitrogen and foam-assisted nitrogen in the fractured-vuggy reservoir [17]. A 2D model was established by Xu to reveal the flow characteristics and the EOR mechanism of foam in fractured-vuggy reservoirs [30]. The design and manufacture of the three-dimensional (3D) visual model of the fractured-vuggy reservoirs were complicated, and related research is rare. A 3D visual model of the fractured-vuggy reservoir was established by Qu, and the flow characteristics of oil, gas, and water in the process of oil displacement were studied systematically and comprehensively [31]. Lu also established a 3D visual model and analyzed the flow characteristics and EOR mechanism of foam flooding in fractured-vuggy reservoirs [32].

In this paper, the model parameters and experimental parameters of the physical simulation are designed according to the similarity criterion. Three kinds of visual models of fractured-vuggy reservoirs with different dimensions were fabricated. The experiments of water flooding, nitrogen gas flooding, and gel foam flooding were carried out in these models. The experimental results revealed the flow rules of water, gas, and foam in the fractured-vuggy structure. The main EOR mechanisms of gas and gel foam in fractured-vuggy reservoirs were analyzed. The EOR effects of different flooding methods were evaluated, and the best injection modes of different flooding methods in fractured-vuggy reservoirs were put forward. It provided a theoretical basis and technical support for the application of gas flooding and gel foam flooding in the Tahe Oilfield and other fractured-vuggy reservoirs.

## 2. Results and Discussion

### 2.1. Experimental Results and Analysis of Physical Simulation in the 1D Visual Models

**Figure 1:**
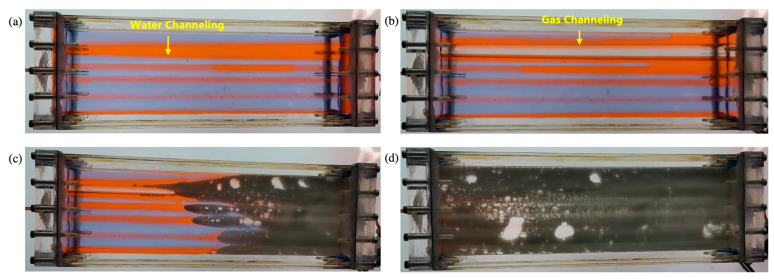
Experiments in the 1D visual model of a single fracture: (**a**) water flooding; (**b**) gas flooding after water flooding; (**c**) gel foam flooding after gas and water flooding; (**d**) experimental result after gel foam flooding.

The flow of viscous fluid conforms to the Boundary Layer theory. The greater the difference in viscosity between the displacement phase and the displaced phase, the more serious the viscous fingering was and the easier it was to form a dominant channeling. The fluid flow in the fracture conforms to the Poiseuille Flow law. During the water flooding process, the flow velocity of fluids at different locations on the cross-section of the fracture was different due to the irregular shape of the fracture and the viscous difference of fluids. The first dominant channeling of water formed at the cross-section where the velocity of fluid was fastest. After that, the flow resistance in the first dominant channeling became the flow resistance of water and remained unchanged. The other places on the cross-section of the fracture were swept by subsequent injection water and gradually formed multiple water channels. After the dominant channelings were formed, all the injected water flowed along the dominant channelings, as shown in Figure S1a and Figure 1a. The flow velocity in the undisplaced area on the cross-section of the fracture was 0.

The viscous fingering was more serious during gas flooding than water flooding because the viscosity ratio between gas and oil is much lower than that between oil and water. After a dominant channeling of gas was formed, the injected gas flowed along the dominant channeling preferentially. It was difficult for the gas to displace residual oil in the cross-section of the fracture at a constant flow rate. Finally, only one dominant channeling of gas was formed in the 1D visual model, as shown in Figure S2a. The gas flooding experiment after water flooding revealed that the injected gas did not necessarily flow along the dominant channeling of water but formed a new dominant channeling, as shown in Figure 1b. This phenomenon was caused by the difference in fluid viscosity and fracture shape.

Since the viscosity of gel foam is much greater than that of water and gas, gel foam displaced all the oil on the cross-section of the fracture, as shown in Figure 1d. All subsequent experiments revealed that gel foam can control fluid mobility in fractures, such as gel foam flooding after water flooding (see Figure S1b), gel foam flooding after gas flooding (see Figure S2b), and gel foam flooding after water flooding and gas flooding (see Figure 1c). Gel foam can uniformly displace fractures with irregular cross-sections, expand the swept volume, and enhance the oil recovery of fractures.

### 2.2. Experimental Results and Analysis of Physical Simulation in the 2D Visual Models

#### 2.2.1. Experimental Results of the 2D Visual Model of Complex Fractures

**Figure 2:**
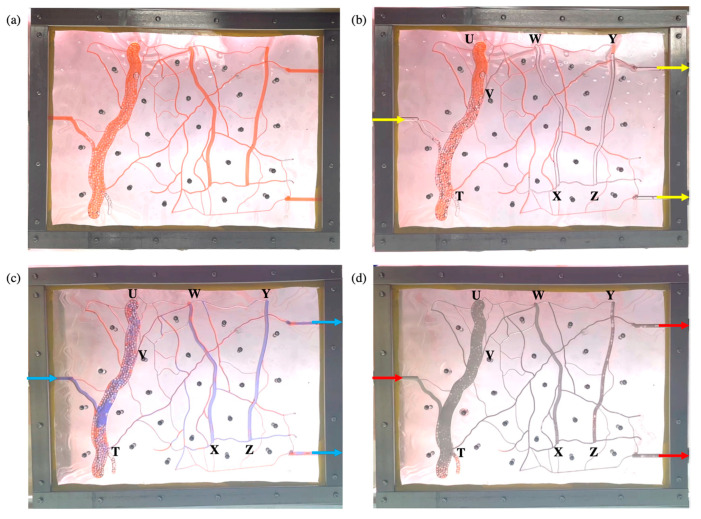
Experimental results of the 2D visual model of complex fractures: (**a**) saturated with oil; (**b**) gas flooding; (**c**) water flooding; (**d**) gel foam flooding.

Water flooding, gas flooding, and gel foam flooding experiments were carried out in the 2D visual model of complex fractures, and the experimental results are shown in Figure 2. Compared to water flooding, the large viscosity difference between gas and oil, as well as the large oil-gas interfacial tension, lead to the extremely poor microscopic displacement efficiency of gas flooding in fractures. The injected gas rapidly broke through along the porous section in the large fracture (U–T). Then, it successively migrated along the fracture (V–W), the fracture (W–X), the fracture (X–Z), and the fracture (Z–Y). Finally, the injected gas formed a channel from the inlet to the outlet, and the result of the gas flooding experiment is shown in Figure 2b. Gas flooding with a recovery ratio of 39% did not sweep the small fracture (0.5 mm) in the 2D visual model of complex fractures.

The interfacial tension and viscosity difference between water and oil are smaller than those between gas and oil, resulting in the displacement effect of water flooding in the porous section of the large fracture (U–T) media, which is much better than gas drive. After the injected water swept the fracture (U–T), it migrated to the fracture (W–X) along small fractures (1 mm). Then, the subsequent water swept through fractures (0.5 mm and 1 mm) between fracture (W–X) and fracture (Z–Y). Finally, injected water migrated to the outlet along the fracture (Z–Y). Water flooding with a recovery ratio of 68% swept some of the small fractures between the large fractures, as shown in Figure 2c.

The larger viscosity difference and lowest interfacial tension between gel foam and oil compared to the others enable the gel foam to sweep most of the connected fractures in this model. The small fractures (0.5 mm and 1 mm) that cannot be swept by the water or gas can be effectively displaced by gel foam. Gel foam flooding with a recovery ratio of 89% is shown in Figure 2d. The residual oil after gel foam flooding mainly existed in the fractures with blind ends.

#### 2.2.2. Experimental Results of the 2D Visual Model of Complex Vugs

**Figure 3:**
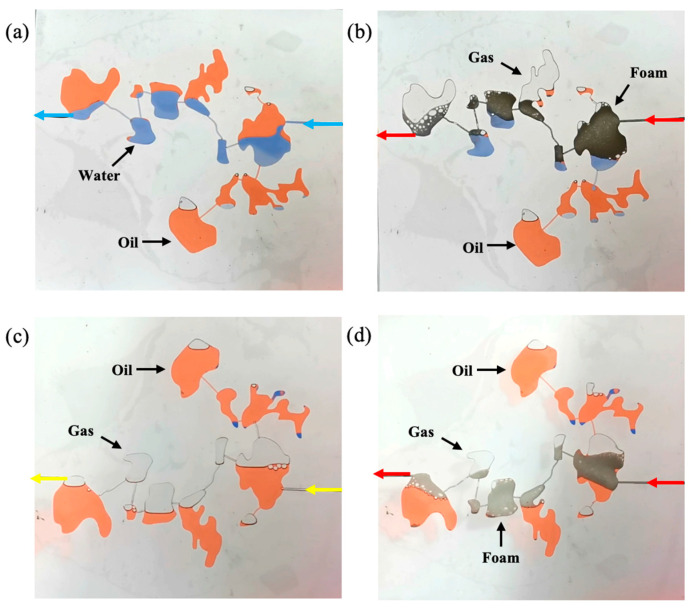
Experimental results of the 2D visual unfilled model of complex vugs: (**a**) water flooding; (**b**) water flooding after gas flooding; (**c**) gas flooding; (**d**) gel foam flooding after gas flooding.

Water flooding, gas flooding, and gel foam flooding experiments were carried out in the 2D visual model of complex fractures, and the experiment result of the unfilled model is shown in Figure 3. The results indicated that the gravitational differentiation was obvious during the water flooding, gas flooding, and gel foam flooding process in the fracture-vuggy structure. Gas flowed along the upper parts, water flowed along the lower parts, and gel foam flowed along the middle parts of the fracture-vuggy structure. Some of the gel foam released gas when it burst in vugs. Then, the gas and water stratified under the influence of gravitational differentiation. Gas migrated upward to replace the oil in the upper vugs and formed a gas cup (see Figure 3b). The final recovery ratio of water flooding was 34.8% (see Figure 3a), the recovery ratio of gel foam flooding after water flooding was 68.2% (see Figure 3b), the recovery ratio of gas flooding was 38.5% (see Figure 3c), and the recovery ratio of gel foam flooding after gas flooding was 54.8% (see Figure 3d).

**Figure 4:**
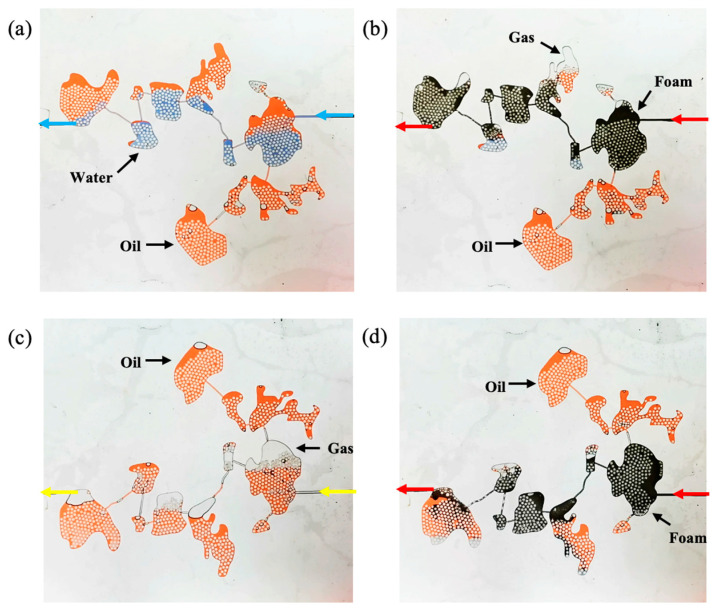
Experimental results of the 2D visual filled model of complex vugs: (**a**) water flooding; (**b**) water flooding after gas flooding; (**c**) gas flooding; (**d**) gel foam flooding after gas flooding.

The experiment result of the filled model is shown in Figure 4. The results indicated that the swept volume of water flooding and gel foam flooding in filled vugs was larger than that of unfilled vugs. The swept volume of gas flooding was just the opposite. Due to the difference in fluid density, gas was extremely easy to channel in filled vugs, but gel foam could control the mobility very well. The final recovery ratio of water flooding was 45.8% (see Figure 4a), and the recovery ratio of gel foam flooding after water flooding was 66.7% (see Figure 4b). The final recovery ratio of gas flooding was 31.2% (see Figure 4c), and the recovery ratio of gel foam flooding after gas flooding was 58.9% (see Figure 4d).

### 2.3. Experimental Results and Analysis of Physical Simulation in the 3D Visual Model

The TK671 well-group unit is a typical epikarst reservoir with complex connection relationships. This unit has six wells, all of which are located in connected vugs. Since the TK661 well is located at the bottom of the vug and is the deepest of all wells, the TK661 well was selected as the water injection well.

#### 2.3.1. Gas Flooding and Gel Foam Flooding in TK692X

**Figure 5:**
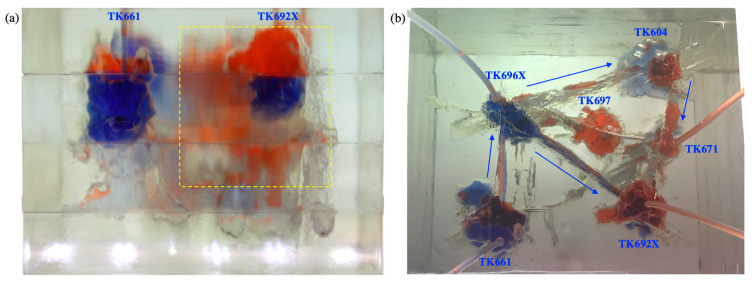
Experimental results of water flooding in the 3D visual model: (**a**) the front view and (**b**) the overhead view.

Flooding experiments were carried out in the 3D visual model. The TK661 well was chosen to be a water injection well at first. The TK692X well was chosen to be a gas injection well or gel foam injection well. The TK661 well, TK696X well, and TK671 well were chosen to be production wells. After the water was injected into the TK661 well, it displaced the residual oil in the bottom vug and then migrated to other vugs through the fractures connected with the bottom vug. The injected water flowed forward along the main channel (TK661-TK696X-TK604-TK671). Under the influence of gravity, water displaced the oil in the lower part of the model, which led to an increase in the oil–water interface. The water effectively swept the large fracture channel between the TK696X well and the TK692X well but did not sweep other flow channels (see Figure 5b). The experiment of water flooding was stopped when water was produced from the TK696X well at the lower part of the model. The complex structure of the fracture-vugs reservoir leads to an inconsistent height of the oil–water interface. After water channeling occurred in the model, the oil–water interface was high in the swept vugs and low in a large number of unswept vugs (see Figure 5a). Water flooding (with a recovery ratio of 29%) swept the residual oil in large fractures and the lower part of the vugs, but it cannot effectively displace the residual oil in other channels, blind-end vugs, and poorly connected vugs. Multiple flow channels were formed during water flooding.

**Figure 6:**
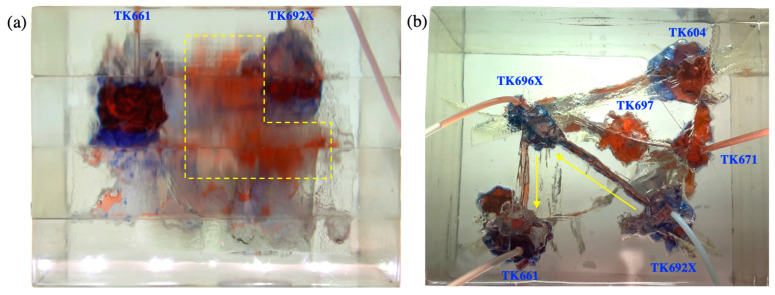
Experimental results of gas flooding in the 3D visual model (gas channeling occurred in TK661 well): (**a**) the front view and (**b**) the overhead view.

**Figure 7:**
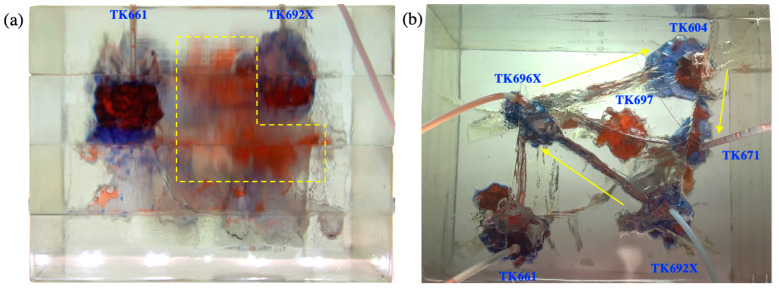
Experimental results of gas flooding in the 3D visual model (gas channeling occurred in TK671 well): (**a**) the front view and (**b**) the overhead view.

Gas was injected into the TK692X well after water flooding. It displaced the residual oil of the vug under the TK962X well and then broke through along the large fracture between the TK692X well and the TK696X well (see Figure 6a). After that, gas swept the vug under the TK966X well and then migrated along the fracture to the TK611 well. Gas was produced from the TK611 well and formed a gas channeling in the model (see Figure 6b). The well shut-in measure was taken for the TK661 well, which led to the subsequent gas beginning to migrate along another fracture channel (TK696X-TK604-TK671). The residual oil at the top of the TK604 well and the TK671 well was gradually displaced by gas. Finally, a gas channeling occurred in the TK671 well (see Figure 7). Under the influence of gravity, gas flooding swept the large fractures and vugs at the upper part of the model but could not effectively displace the residual oil in the fractures and vugs at the lower part of the model. Gas flooding, with a recovery ratio of 56%, was 27% higher than water flooding. Only one flow channeling was formed during gas flooding.

**Figure 8:**
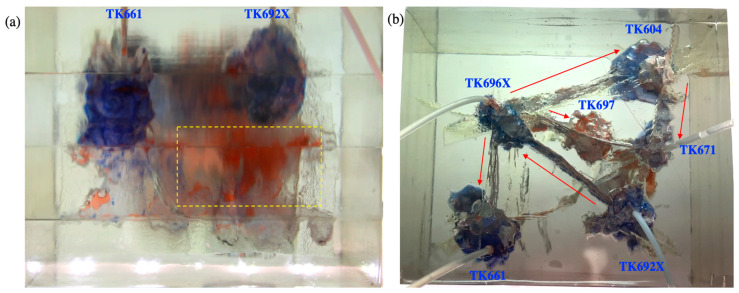
Experimental results of gel foam flooding in the 3D visual model: (**a**) the front view and (**b**) the overhead view.

**Figure 9:**
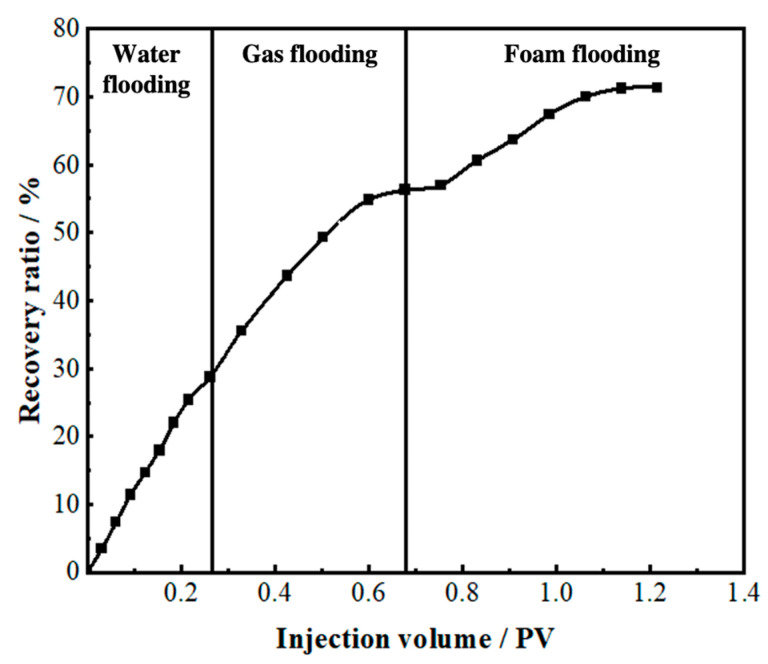
The recovery ratio of the 3D visual model (injection well: TK692X well).

Gel foam flooding was carried out in the model after gas flooding. After the gel foam was injected into the TK692X well, the vug at the bottom of the TK692X well was filled by gel foam, and all remaining oil in it was displaced (see Figure 8a). Gel foam migrated along the large fracture between the TK692X well and the TK696X well and effectively displaced the residual oil in the vug at the bottom of the TK696X well. After that, the gel foam moved at a constant speed in three channels (TK696X-TK604, TK696X-TK697, and TK696X-TK661) at the same time, as shown in Figure 8b. The vugs at the bottom of the TK604 well and the TK661 well were effectively displaced by gel foam. The residual oil in the vug at the bottom of the TK697 well has not been completely displaced by gel foam because of its complex structure. The experiment of gel foam flooding was stopped after gel foam swept the vug at the bottom of the TK671 well. Gel foam has an excellent capability of mobility control and high microscopic displacement efficiency, which can uniformly sweep most of the connected channels and effectively displace the residual oil. Gel foam flooding, with a recovery ratio of 71.5%, was 15.5% higher than gas flooding (see Figure 9).

#### 2.3.2. Gas Flooding and Gel Foam Flooding in TK671

Flooding experiments were carried out in the 3D visual model. The TK661 well was chosen to be a water injection well at first. The TK671 well was chosen to be a gas injection well or gel foam injection well. The TK661 well, TK696X well, and TK692X well were chosen to be production wells.

During the gas flooding process, gas displaced the residual oil at the top of the vug under the TK671 well and then broke through along the main channel (TK671-TK604-TK696X-TK661). The residual oil at the top of the fractured-vuggy structure on this channel was swept by gas, and the gas channeling finally occurred in the TK661 well. Gas flooding did not sweep the fractured-vuggy structure on other channels (TK692X-TKTK696X and TK671-TK697-TKTK696X). The experimental result of gas flooding is shown in Figure S3, and the recovery ratio of gas flooding was 52.1%.

**Figure 10:**
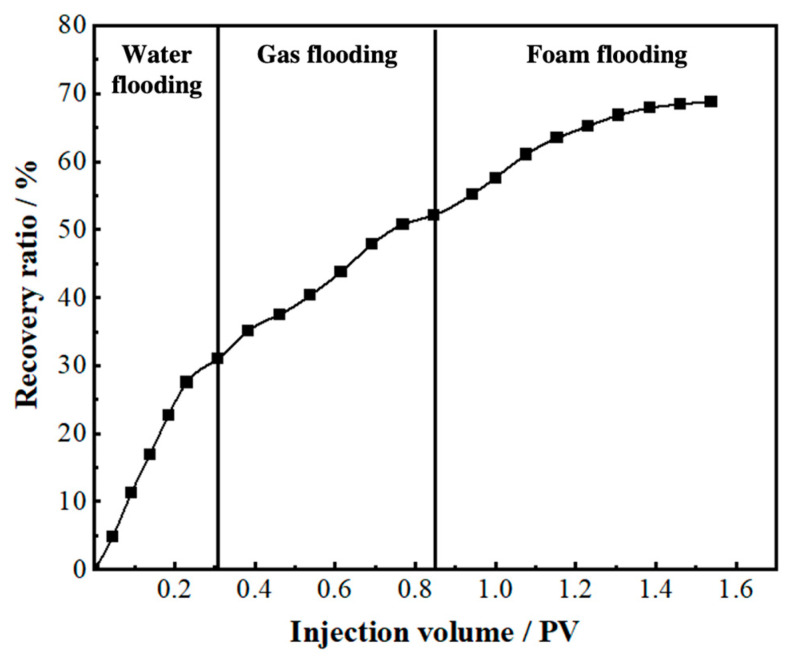
The recovery ratio of the 3D visual model (injection well: TK671 well).

During the gel foam flooding process, gel foam not only displaced the main channel (TK671-TK604-TK696X-TK661) effectively but also swept a new flow channel (TKTK696X-TK692X). However, due to the poor connectivity of the vug under the TK697 well, the gel foam was unable to sweep the residual oil in this vug (see Figure S4). Gel foam flooding with a recovery ratio of 69.7% is shown in Figure 10.

#### 2.3.3. Gas Flooding and Gel Foam Flooding in TK697

Flooding experiments were carried out in the 3D visual model. The TK661 well was chosen to be a water injection well at first. The TK697 well was chosen to be a gas injection well or gel foam injection well. The TK661 well, TK671 well, and TK692X well were chosen to be production wells.

During the gas flooding process, gas displaced the residual oil at the top of the vug under the TK697 well and then broke through along a flow channel (TK697-TK696X-TK661). Gas channeling finally occurred in the TK661 well. Gas flooding did not sweep the fractured-vuggy structure on other channels (TK692X-TKTK696X, TK692X-TK604-TK671, and TK671-TK697). The experimental result of gas flooding is shown in Figure S5, and the recovery ratio of gas flooding was 53%.

**Figure 11:**
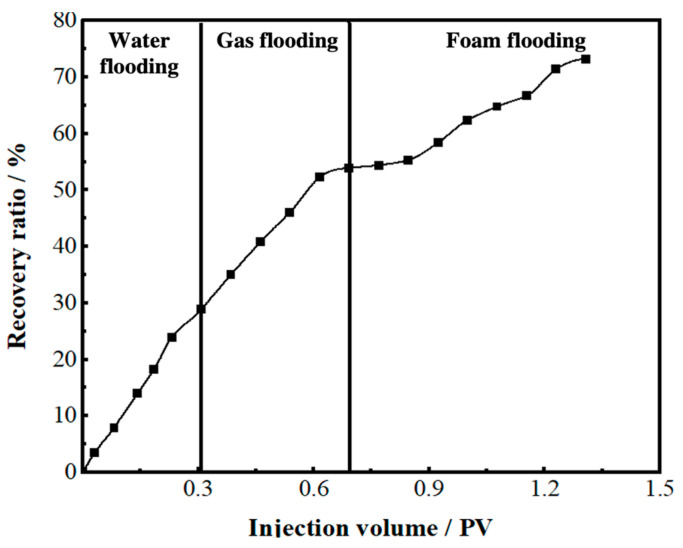
The recovery ratio of the 3D visual model (injection well: TK697 well).

During the gel foam flooding process, the gel foam uniformly swept the fractures and vugs around the TK697 well and depressed the oil-gas interface near the bottom of the TK697 well. The residual oil submerged by the bottom water and that could not be swept by water or gas was effectively displaced by gel foam. Compared with gas flooding, gel foam flooding finally swept two new channels (TK696X-TK692X and TK696X-TK604-TK671), as shown in Figure S6. Gel foam flooding with a recovery ratio of 73.3% is shown in Figure 11.

The experimental results of the 3D visual model indicated that injected water flowed along the lower part of the fractured-vuggy reservoir and could form multiple flow channels. Gas flows along the upper part of the fractured-vuggy reservoir and usually forms only one flow channel. The complex fractured-vuggy structure led to the disunity difference of the oil–water interface in different vugs. Once the channeling was formed, the residual oil in a large number of fractured-vuggy structures was difficult to replace.

During the development process of fractured-vuggy reservoirs, the well at the bottom of the reservoir should be selected for water injection to prevent some poorly connected vugs from being submerged by water and unable to be swept by subsequent displacement media. The well located in a large vug at the upper part of the reservoir should be selected for gas injection to better displace the attic oil at the top of the vugs. Gel foam flooding flowed uniformly in different directions. Therefore, gel foam flooding has no special requirements for the location of the injection well but has higher requirements for the stability and viscosity of gel foam. Gel foam with strong stability and high viscosity might have a better displacement effect in the fractured-vuggy reservoir.

### 2.4. EOR Mechanism of Gel Foam in Fractured-Vuggy Carbonate Reservoirs

The capillary number is a dimensionless number that represents the ratio of viscous force to capillary force in the oil phase. It can reflect the displacement effect in porous media. It can be seen from the formula that the displacement effect of displacement medium on fractures is affected by interfacial tension, viscosity, and flow velocity. The decrease in the interfacial tension between the displacement phase and the displaced phase, the increase in the viscosity of the displacement phase, and the increase of the displacing velocity all have a positive effect on the sweep efficiency. In fractured-vuggy reservoirs, the interfacial tension and fluid viscosity of gas (except CO_2_) and water are constant, and the recovery ratio can be improved by increasing velocity. Gel foam can improve the mobility ratio by increasing its viscosity, and the surfactant contained in the gel foam can reduce the interfacial tension between oil and water. Thus, gel foam has a better potential to improve oil recovery.

(1) $$N_{c}=\frac{v\mu }{\sigma }$$

where the $N_{c}$ is the capillary number; the *v* is the flow velocity of displacement media, m/s; *µ* is the viscosity of displacement media, mPa·s; and the σ is the interfacial tension between displacement media and displaced media, mN/m.

#### 2.4.1. Reducing Interfacial Tension

**Figure 12:**
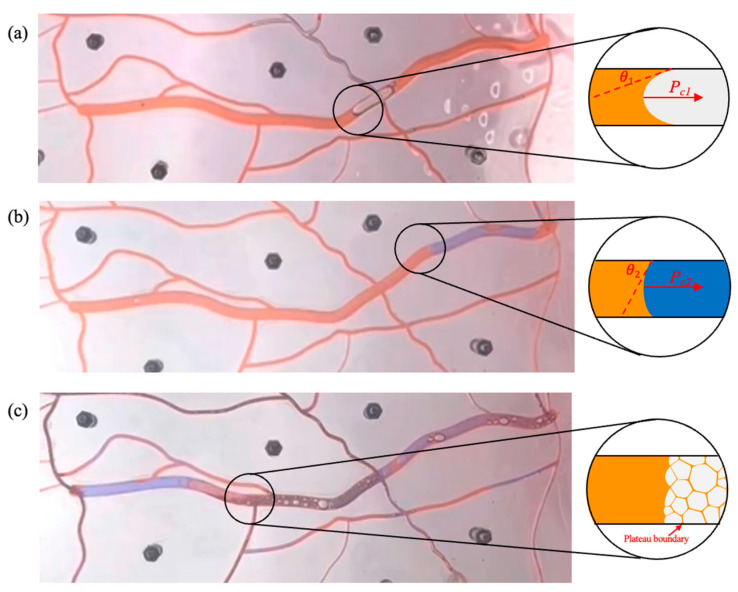
The sweep process of gas flooding, water flooding, and gel foam flooding in fractures. (**a**) the sweep process of gas flooding, (**b**) the sweep process of water flooding and (**c**) the sweep process of foam flooding. (*θ*_1_ = 18°, $\theta_{2}$ = 58°, $r_{1}$ = 1.5 mm, $r_{2}$ = 2.6 mm, $\sigma_{1}\;$= 27.49 mN/m, $\sigma_{2}\;$= 21.75 mN/m, Then $P_{c1}$ ≈ 34.86 Pa, $P_{c2}$ ≈ 8.87 Pa).

The surfactant in gel foam can reduce the interfacial tension between oil and water and improve the displacement efficiency of gel foam in the fractured-vuggy structure. It can be seen from Figure 12 and Table S1 that *P_c_*_1_ was larger than *P_c_*_2_. The resistance that needed to be overcome when gas entered the fracture (*P_c_*_1_) was larger than that when water entered the fracture (*P_c_*_2_). Thus, water could sweep some small fractures that could not be swept by gas. The displacement front of gel foam flooding was formed by a large number of small-size gel foam in fractures and vugs. A plateau boundary formed between gel foam and rock, but there was no obvious two-phase interface at the displacement front. There was no two-phase boundary, and the resistance that needed to be overcome when gel foam entered the fracture was far less than that of water and gas. Therefore, gel foam could sweep most of the fractures that could not be swept by gas or water. At the same time, the surfactant in the gel foam changed the wettability of the rock and effectively peeled off the oil film in the fractured-vuggy structure. The displacement effect of different displacement media is shown in Figures S7 and S8.

(2) $$P_{c}=\frac{2\sigma \cos\theta }{r}$$

where the *P_c_* is the capillary force, mPa; the σ is interfacial tension, mN/m; the θ is the contact angle, °; and the *r* is the capillary radius, m.

#### 2.4.2. Increasing Mobility Ratio

Formula 3 shows that viscosity and permeability determine the mobility of the fluid in the fractured-vuggy structure. The smaller the mobility ratio is, the more stable the displacement front of the fluid in the fracture-vuggy structure is, and the larger the swept volume is. During the experiment, the viscosity of gel foam was much higher than that of water and gas, and the mobility of gel foam flooding was much lower than that of gas flooding and water flooding. Gel foam effectively restrained viscous fingering and stabilized the displacement front. In the filling medium of the fractured-vuggy structure, the gel foam was greatly affected by the Jamin effect, resulting in the permeability of the gel foam being much less than that of oil and water. As shown in Figures S7 and S8, the gel foam had a good sweep effect in the fractured-vuggy reservoir with varying apertures, different sizes, and different filling degrees.

(3) $$M=\frac{\lambda_{D}}{\lambda_{d}}=\frac{K_{D}\mu_{d}}{K_{d}\mu_{D}}$$

where the $\lambda_{D}$ is the mobility of the displacement phase, the $K_{D}$ is the permeability of the displacement phase, mD; the $\mu_{D}$ is the viscosity of the displacement phase, mPa·s; the $\lambda_{d}$ is the mobility of the displaced phase, the $K_{d}$ is the permeability of the displaced phase, mD; the $\mu_{d}$ is the viscosity of the displaced phase, mPa·s.

#### 2.4.3. Selectively Plugging High Permeability Channels

It was found that gel foam has the ability to selectively plug high permeability channels and start small fracture channels so as to achieve the effect of expanding the swept volume. A gas channeling was formed in the large fracture (WX), and the residual oil in small fractures could not be swept by the subsequent fluid, as shown in Figure S9a. After gel foam entered the large fracture (WX), the flow resistance in the large fracture (WX) was increased, and gel foam began to enter the small fracture (RS), as shown in Figure S9b. After the large fracture (WX) was filled with gel foam, the small fracture (RS) was effectively swept by the subsequent gel foam, as shown in Figure S9c. Gel foam played an effective role in plugging high permeability fracture channels in fractured-vuggy reservoirs.

#### 2.4.4. Discontinuous Flow

The microscopic velocity of the continuous phase is the same, but the microscopic velocity of the discontinuous phase is different between different phases during the flow process. This difference in velocity can lead to a fluctuation of the flow field and has a positive effect on expanding the swept volume. As shown in Figure S10, gas and water were continuous phases. Gas flowed in a continuous state in both the filled fractured-vuggy structure and the unfilled fractured-vuggy structure because of its low viscosity. The viscosity of water is usually higher than that of gas but less than that of oil. When the viscosity difference between oil and water was small, it flowed in a discontinuous state in the filling fractured-vuggy structure but in a continuous state in the unfilled fractured-vuggy structure. Gel foam is a kind of discontinuous fluid that flows in a discontinuous state when flowing in both the filled fractured-vuggy structure and the unfilled fractured-vuggy structure. In addition, the change in viscosity and size was beneficial to the discontinuous flow of gel foam.

The viscous resistance of gel foam is usually characterized by apparent viscosity. There is a positive correlation between gel foam quality and apparent viscosity [33]. In the process of flow, the adsorption loss of surfactant and the increase of gel foam liquid phase will lead to the decrease of gel foam quality. The change in gel foam quality will lead to a change in the apparent viscosity of gel foam in different positions and then affect the flow velocity of gel foam at different positions, resulting in fluctuations in the flow field.

In porous media, the flow velocity of gel foam is negatively related to the size of the gel foam. The smaller the size of the gel foam is, the faster the flow velocity is [33]. The coarsening of gel foam is inevitable, resulting in a gradual increase in the size of the gel foam [34]. In addition, gel foam is easy to burst in the presence of oil. Under the influence of multiple factors, the size of the gel foam gradually varies in different positions in the reservoir. Due to the superposition of the Jamin Effect, the velocity of the gel foam in different locations will be different, which will also result in fluctuations in the flow field.

## 3. Conclusions

In this study, the visual and physical models of a fractured-vuggy reservoir were designed and fabricated innovatively based on the real reservoir geological data. The experimental study on improving oil recovery by water, gas, and gel foam flooding was carried out in these models. Combined with the displacement experiments from one-dimensional to three-dimensional, the following results and conclusions are obtained.

1. Water has multiple flow channels when flowing in the fractured-vuggy reservoir, but the recovery ratio of water flooding is limited. Gas channeling with a single flow channel is easy to form in fractures and vugs, and the EOR of gas flooding is unsatisfactory. Gel foam flooding with an excellent capability of mobility control and a high microscopic displacement efficiency has great potential to enhance oil recovery in fracture-cave reservoirs;
2. The EOR mechanisms of gel foam in fractured-vuggy reservoirs are mainly as follows: reducing interfacial tension, increasing mobility ratio, selectively plugging high permeability channels, and discontinuous flow;
3. By injecting water from the well at the bottom of the reservoir and gas from the well located in the vug at the high part of the reservoir, multiple technologies can be used together to enhance oil recovery;
4. Gel foam has no special restriction on the location of the injection well, but gel foam with strong stability and high viscosity should be selected in fractured-vuggy reservoirs.

## 4. Physical Model Design and Fabrication

In this study, a visual, physical simulation method is used to study the flooding behavior and sweep mechanism of gas flooding and gel foam flooding in fractured-vuggy carbonate reservoirs (as shown in Table 2). The physical models, including the 1D visual model, the 2D visual model, and the 3D visual model, were designed based on similarity criteria, as well as the experimental parameters. The matrix permeability of fractured-vuggy carbonate reservoirs is extremely low, which leads to poor flow ability of fluid in the matrix. The main flow channel of fluid is the fractured-vuggy structure, and the fluid motion in the fractured-vuggy structure conforms to Newton’s law and the laws of thermodynamics. The fluid flow was similar as long as the various influencing factors in the fluid motion equation were similar [35]. To make the fluid flow pattern in the physical model of the fractures and vugs similar to that in the actual reservoir, the characteristic parameters of fractures and cavities are designed by geometric similarity, and the physical properties of fluids and injection parameters are designed by kinematic similarity and dynamic similarity in this study (as shown in Table 3). Combined with field production data in the Tahe Oilfield, the design of the kinematic similarity and dynamic similarity was carried out for the physical properties of the fluid, production pressure differences, and injection velocities.

According to the similarity coefficient, the similarity number $F_{Q}=\frac{Q}{r^{2}u}=0.99$ and $F_{G}=\frac{\Delta p}{\rho_{0}gB}=1.06$ were obtained. It was proved that the actual fractures in reservoirs and the laboratorial physical fracture model satisfy the above similarity criteria.

Note: $F_{Q}$ is kinematic similarity; Q is injection rate, $m^{3}\cdot d^{-1}$; r is wellbore diameter, mm; u is flow rate, $m\cdot s^{-1}$; $F_{G}$ is dynamic similarity; Δp is the pressure difference, kPa; $\rho_{0}$ is the oil density, $g\cdot {\mathrm{cm}}^{-3}$; g is gravitational acceleration, $m\cdot s^{-2}$; *B* is fracture aperture, cm.

### 4.1. One-Dimensional Visual Model of a Single Fracture

**Figure 13:**
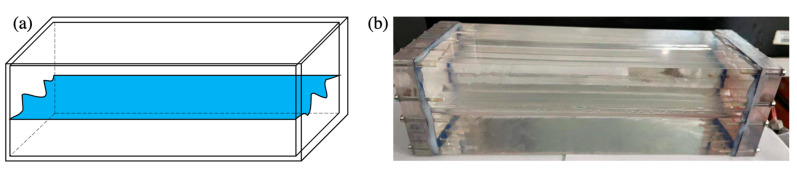
1D visual model of a single fracture: (**a**) design diagram and (**b**) physical diagram.

The 1D visual model of a single fracture with varying apertures was designed to study the flow behavior in wide fractures. The same shape of the fracture was etched on two pieces of plexiglass (with an oleophilic surface) through the laser etching technique based on the design diagram (see Figure 13a). Then, the plexiglass was covered by the other plexiglass and fixed by a steel frame, with a 10 mm distance between the two plates. Finally, the tightness of the 1D visual model is ensured by high-temperature sealing bonding. The 1D visual model of a single fracture with an apparent size of 30 cm × 10 cm × 10 cm and a 10 mm height fracture is shown in Figure 13b.

### 4.2. Two-Dimensional Visual Model

Two kinds of 2D visual models were designed in this study, including a 2D visual model of complex fractures and a 2D visual model of complex vugs.

#### 4.2.1. Two-Dimensional Visual Model of Complex Fractures

**Figure 14:**
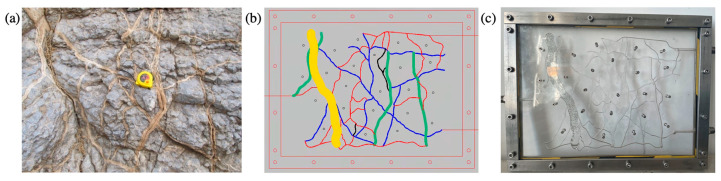
The 2D visual model of complex fractures: (**a**) the photo of the actual fractured-vuggy reservoir, (**b**) the design diagram, (**c**) the physical diagram. The size of the yellow fracture is 10 mm wide and 5 mm deep; the size of the green fracture is 3 mm wide and 3 mm deep; the size of the blue fracture is 1 mm wide and 3 mm deep; the size of the red fracture is 0.5 mm wide and 3 mm deep. The filler used in the model is oleophilic transparent acrylic beads (2 mm).

The 2D visual model of complex fractures was designed according to the photograph of the actual fractured-vuggy reservoir (Figure 14). Based on the model design diagram, the laser etching technique was used to etch the fracture on the plexiglass (oleophilic surface). After etching, another plexiglass was bonded onto it with a high-temperature sealing treatment and then fixed with a steel frame. The 2D visual model of complex fractures with an external dimension of 26 cm × 20 cm × 2 cm is shown in Figure 14c.

#### 4.2.2. Two-Dimensional Visual Model of Complex Vugs

**Figure 15:**
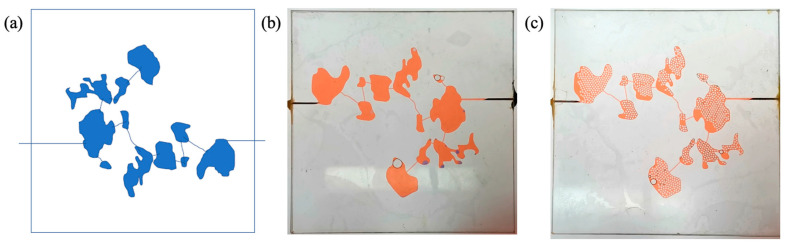
The 2D visual model of complex vugs: (**a**) the design diagram, (**b**) the physical diagram of the unfilled model, (**c**) the physical diagram of the filled model. The width of the fracture is 1 mm, and the depth of fractures and vugs is 3 mm in this model. The filler used in the model is oleophilic transparent acrylic beads (2 mm).

In the same way as the 2D visual model of complex fractures, the 2D visual model of complex vugs with an external dimension of 10 cm × 10 cm × 2 cm was produced through the laser etching technique. In addition, an unfilled model (Figure 15b) and a filled model (Figure 15c) were designed and fabricated to characterize the different filling degrees in vugs.

### 4.3. Three-Dimensional Visual Model of Fractured-Vuggy Carbonate Reservoir

**Figure 16:**
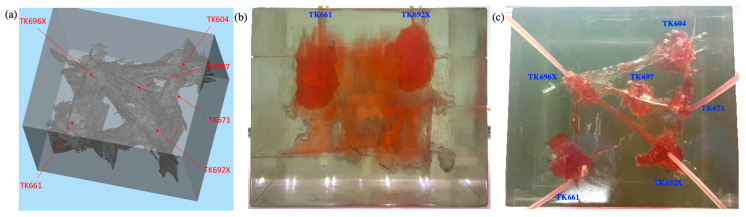
The 3D visual model of the fractured-vuggy carbonate reservoir: (**a**) the 3D design diagram, (**b**) the front view of the physical diagram, (**c**) the overhead view of the physical diagram.

The 3D visual model of the fractured-vuggy carbonate reservoir was made with 3D printing technology. The 3D geological data of fractures and vugs of the TK671 well-group unit in the Tahe Oilfield were selected to design this model. In this 3D geological data, 22 sections (line.1~line.22) were intercepted at equal distances, and 3 over-well sections (line.24~line.26) were intercepted according to the flow condition between wells (see Figures S11 and S12). After the obtained structure data of sections were imported into 3D simulation software and optimized, the 3D visual model of the fractured-vuggy carbonate reservoir was produced using a 3D printer. The 3D visual model of the TK671 well-group unit with an external dimension of 18 cm × 14 cm × 14 cm is shown in Figure 16.

## 5. Experimental Materials and Process

### 5.1. Experimental Materials

The fractured-vuggy carbonate reservoir in the Tahe Oilfield is characterized by high temperature, high pressure, and high mineralization. It does not have a similar capability to foaming by shearing as porous media. For this reason, highly stable gel foam is injected into the Tahe Oilfield after foaming on the ground. This kind of gel foam can be transported stably in the fractured-vuggy reservoir. The chemical reagents used to prepare for highly stable gel foam include the foaming agent (SS-163, Qingdao Changxing High-tech Development Co., Ltd., Qingdao, China), Sodium dodecyl sulfate, α-modified starch, Acrylamide, N,N′-Methylenebisacrylamide, and Potassium persulfate. The preparation method can be referred to in our previous study [12]. The gel foam quality used in this experiment was 70%.

The simulated oil is a mixture of liquid paraffin and kerosene with a viscosity of 60 mPa·s and a density of 0.83 g/mL. The saltwater used in the experiment with a salinity of 250 g/L is prepared with deionized water and inorganic salts. The composition of inorganic salts is Na_2_SO_4_ (417 mg/L), NaHCO_3_ (576 mg/L), NaCl (207,759 mg/L), CaCl_2_ (41,106 mg/L), MgCl_2_ (3549 mg/L). To better observe the experimental results, the simulated oil was dyed red with Sudan red, and the salt water was dyed blue with blue ink. The gas used in the experiment was industrial nitrogen (99.2% purity).

### 5.2. Experimental Instruments

The instruments used to prepare the gel foam liquid included a hydrothermal reactor with high temperature and high pressure, a B75 agitator, and beakers. The instruments used to simulate the core-flooding experiment under reaction temperature and pressure included a high-temperature dryer, a displacement pump, a gas flow controller, a thermostat, a foam generator, a nitrogen cylinder, an intermediate vessel, six-way valves, and connecting pipelines. The working pressure of the displacement pump is 0~20 MPa, and its working flow is 0~20 mL/min. The flow range of the gas flow controller is 0.2~20 mL/min, its working pressure difference is 0.1~0.4 MPa, and its working pressure is 0~20 MPa. The volume of the intermediate vessel is 2000 mL, and its working pressure is 0~40 MPa.

### 5.3. Experimental Process

**Figure 17:**
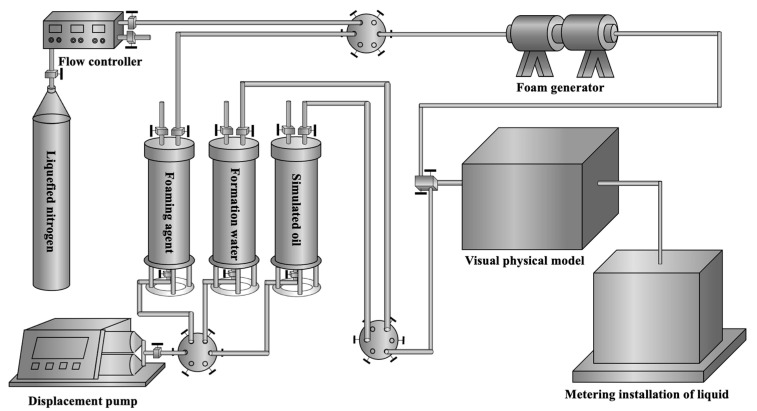
The flow chart of visual models.

The 1D visual model of a single fracture, the 2D visual model, and the 3D visual model of the fractured-vuggy carbonate reservoir were connected according to the flow chart (Figure 17). The experimental steps are as follows: a. Oil was saturated into the visual models, and the saturation volume was recorded. b. Water (gas/foam) was injected into the model at a rate of 4 mL/min. The oil production and the experimental process were recorded. c. The experiment was stopped when the oil production was 0 mL/min.

During the experiments, the 1D visual model of a single fracture, the 2D visual model of complex fractures, and the 3D visual model of the fractured-vuggy carbonate reservoir were placed horizontally, and the 2D visual model of complex vugs was placed vertically.

## Figures and Tables

**Table 1:** A brief review of visual models of fractured-vuggy reservoirs.

Dimension	Model	Materials	Flooding	Size	Ref.
1D	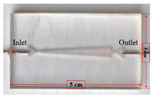	Plexiglass	Nitrogen gas flooding and foam flooding	3 cm × 5 cm	[15]
1D	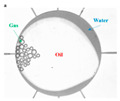	Photopolymer Vero Clear and Fullcure 706	Water flooding and nitrogen gas flooding	340 mm × 340 mm × 200 mm	[26]
2D	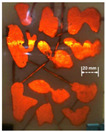	Quartz sand, calcium carbonate and epoxy resin	Water flooding and nitrogen gas flooding	150 mm × 120 mm × 20 mm	[27]
2D	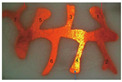	Quartz sand, calcium carbonate, and epoxy resin	Foam flooding	15 cm × 7 cm × 0.68 cm	[28]
2D	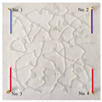	Glass	Nitrogen gas flooding	25 cm × 25 cm	[29]
2D	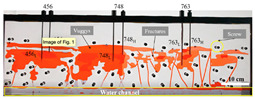	Plexiglass	Nitrogen gas flooding and foam flooding	480 mm × 480 mm × 10 mm	[17]
2D	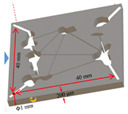	Plexiglass	Foam flooding	40 mm × 40 mm × 1 mm	[30]
3D	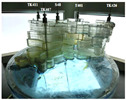	Polymethyl methacrylate	Water flooding and nitrogen gas flooding	1571.29 cm^3^	[31]
3D	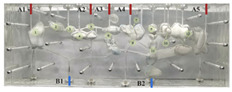	Plexiglass	Water flooding	80 cm × 30 cm × 20 cm	[32]

**Table 2:** Visual models of fractured-vuggy reservoirs in this study.

Models	Materials	Size	Features	Flooding
One-dimensional visual model	Plexiglass	30 cm × 10 cm × 10 cm	Fracture model with irregular cross-section.	Water flooding, gas flooding, and foam flooding
Two-dimensional visual model	Plexiglass	26 cm × 20 cm × 2 cm; 10 cm × 10 cm × 2 cm	Filled fracture model with multi-scale fracture network.	Water flooding, gas flooding, and foam flooding
Three-dimensional visual model	Curable resin	18 cm × 14 cm × 14 cm	The fracture-vuggy reservoir model with complex fracture-vuggy structure and various connectivity.	Water flooding, gas flooding, and foam flooding

**Table 3:** The fracture parameters and similarity coefficients of the laboratorial physical model and actual fractures in reservoirs.

Parameter Source	Pressure Difference (kPa)	Fracture Aperture (mm)	Oil Density (g·cm^−3^)	Gravitational Acceleration (m·s^−2^)	Flow Rate (m·s^−1^)	Injection Rate (m^3^·d^−1^)	Wellbore Diameter (mm)
Reservoirs	2000–14,000	40–2500	0.92	9.8	0.0147–0.147	10–50	120
Laboratory	9.2–34.01	0.2–10	0.821	9.8	0.007–0.049	0.0015–0.002	2
Similarity coefficients	217–411	200–250	1.1	1	2.1–3	6666.7–25,000	60

## Data Availability

Data are available from the corresponding author upon reasonable request.

## Supplementary Materials

The following supporting information can be downloaded at: https://www.mdpi.com/article/10.3390/gels9090722/s1, Figure S1: Experiments results of the 1D visual model of a single fracture: (a) water flooding; (b) water flooding after gel foam flooding; Figure S2: Experiments results of the 1D visual model of a single fracture: (a) water flooding; (b) water flooding after gel foam flooding; Figure S3: Experimental results of gas flooding in the 3D visual model: (a) the front view and (b) the overhead view; Figure S4: Experimental results of gel foam flooding in the 3D visual model: (a) the front view and (b) the overhead view; Figure S5: Experimental results of gel foam flooding in the 3D visual model: (a) the front view and (b) the overhead view; Figure S6: Experimental results of gel foam flooding in the 3D visual model: (a) the front view and (b) the overhead view; Figure S7: The displacement results of gas flooding, water flooding, and gel foam flooding in fractures; Figure S8: The displacement results of gas flooding, water flooding, and gel foam flooding in filled fracture; Figure S9: High permeability fracture channel plugged by gel foam: (a) before gel foam entered the fracture (WX), (b) gel foam entered the fracture (WX), (c) the fracture (WX) plugged by gel foam; Figure S10: The flow behavior of different fluid in fractured-vuggy structure: (a) gas flowed in unfilled model, (b) water flowed in unfilled model, (c) gel foam flowed in unfilled model, (d) gas flowed in filled model, (e) water flowed in filled model, (f) gel foamed flow in filled model. Figure S11: Schematic diagram of the sectional position of the TK671 well-group unit; Figure S12: The over-well sections of the TK671 well-group unit: (a) line.23, (b) line.24, (c) line.25; Table S1: The experimental values of water flooding and gas flooding in fractures.
