# Supplementary material for: Experimental Study on Multi-Dimensional Visualization Simulation of Gas and Gel Foam Flooding in Fractured-Vuggy Reservoirs
Source: Gels. 2023 Sep 6;9(9):722. doi: 10.3390/gels9090722 (PMC10530889; doi:10.3390/gels9090722)
Supplement: Supplementary file 1 [file gels-09-00722-s001.zip › gels-2486986-supplementary.pdf]

Supplementary files

1. Supplementary figures

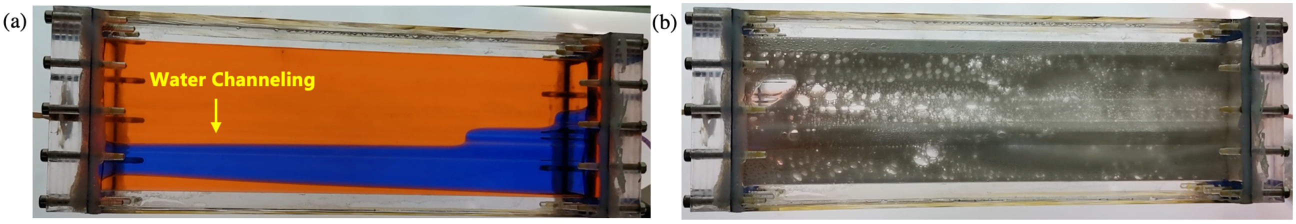

Figure S1. Experiments results of the 1D visual model of a single fracture: (a) water flooding; (b) water flooding after gel foam flooding.

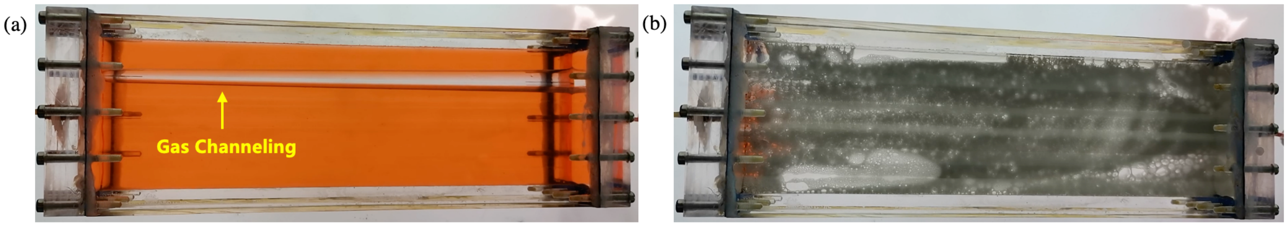

Figure S2. Experiments results of the 1D visual model of a single fracture: (a) gas flooding; (b) gas flooding after gel foam flooding.

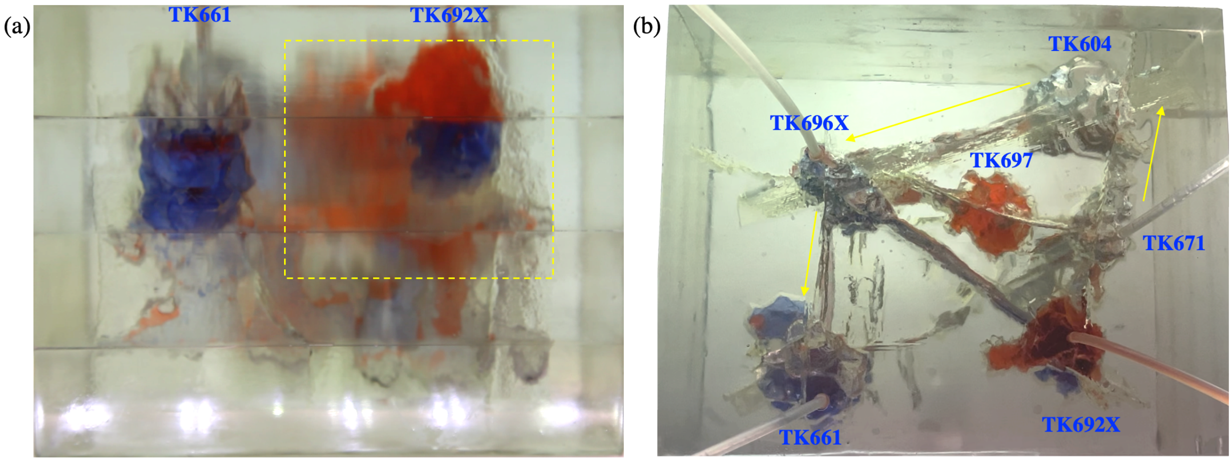

Figure S3. Experimental results of gas flooding in the 3D visual model: (a) the front view and (b) the overhead view.

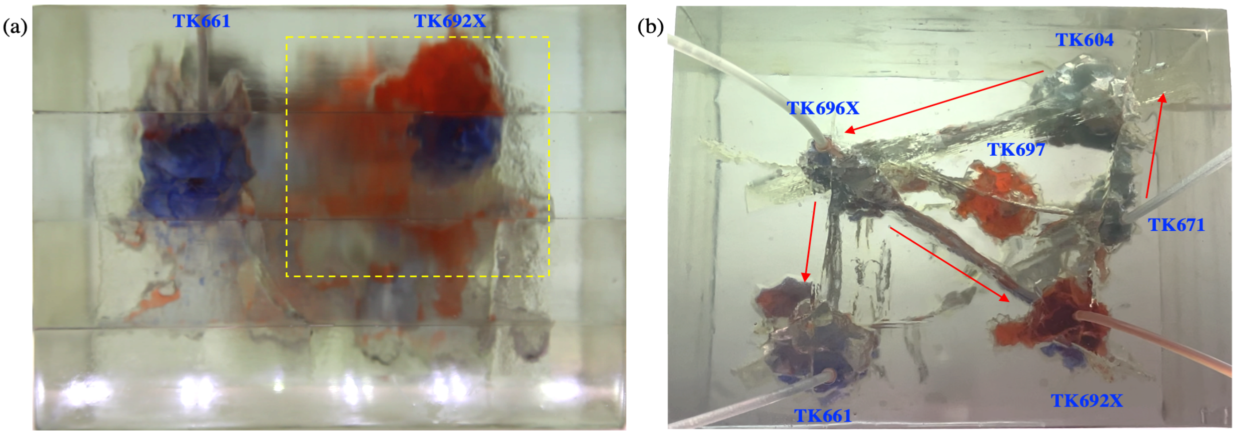

Figure S4. Experimental results of gel foam flooding in the 3D visual model: (a) the front view and (b) the overhead view.

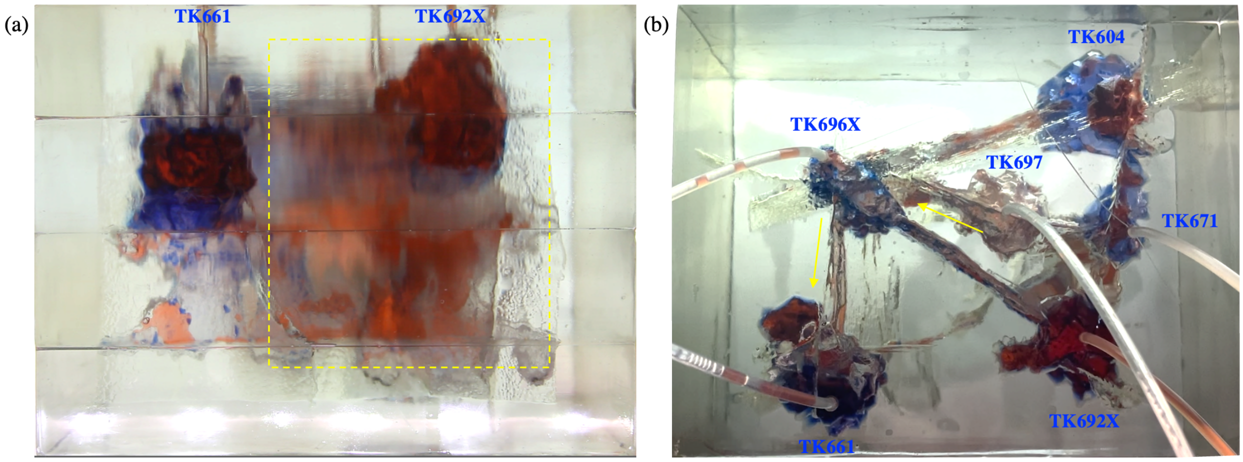

Figure S5. Experimental results of gas flooding in the 3D visual model: (a) the front view and (b) the overhead view.

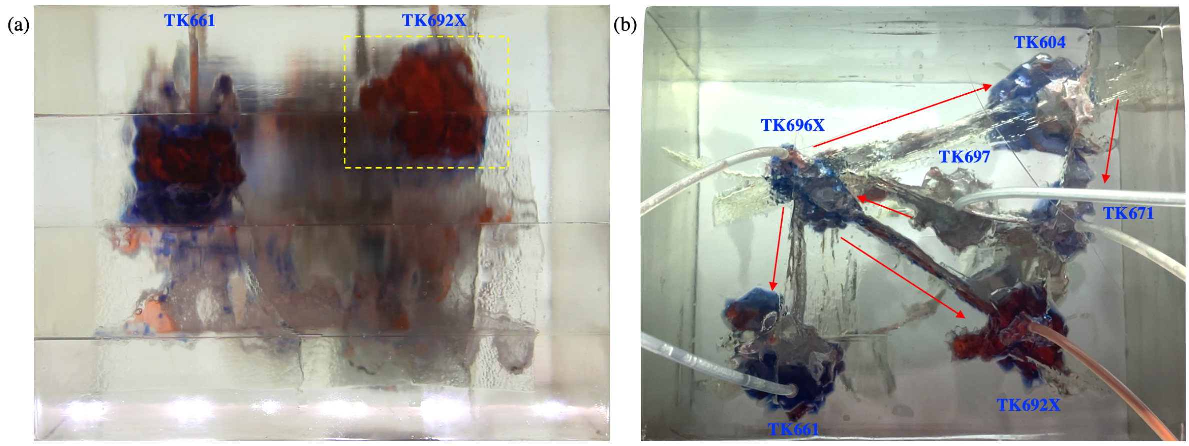

Figure S6. Experimental results of gel foam flooding in the 3D visual model: (a) the front view and (b) the overhead view.

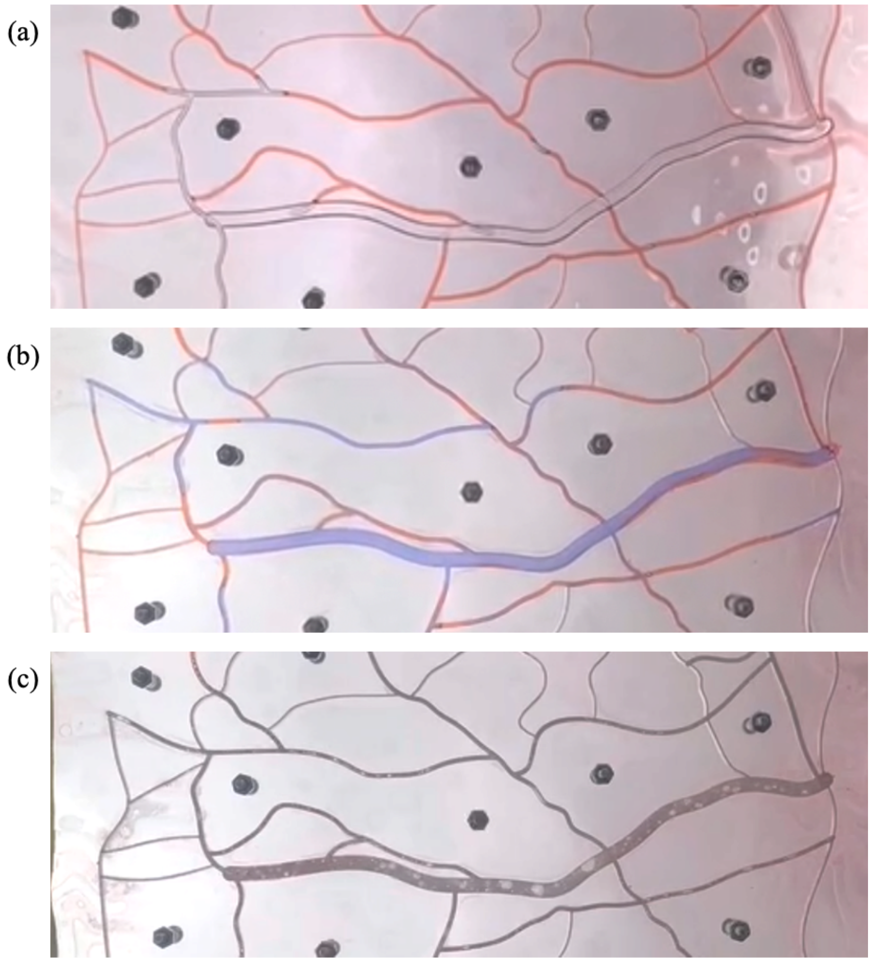

Figure S7. The displacement results of gas flooding, water flooding, and gel foam flooding in fractures.

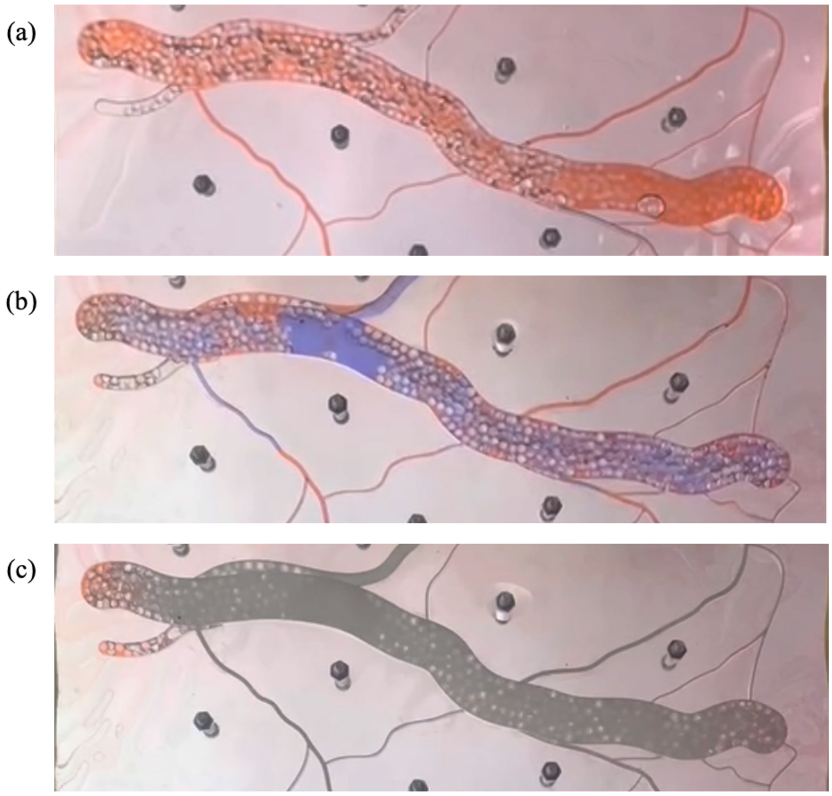

Figure S8. The displacement results of gas flooding, water flooding, and gel foam flooding in filled fracture.

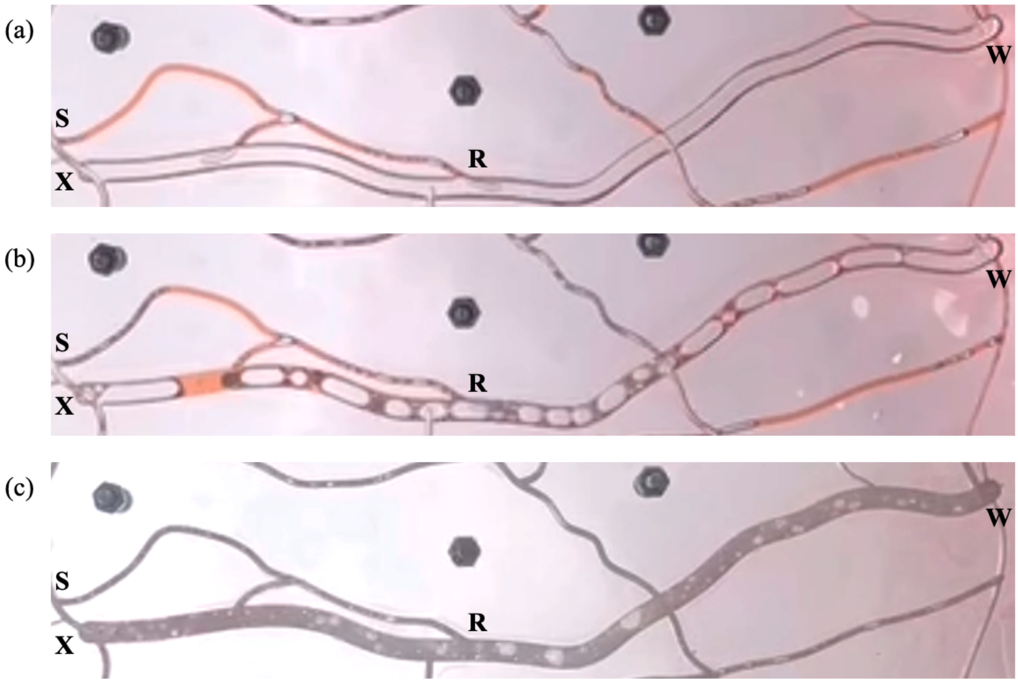

Figure S9. High permeability fracture channel plugged by gel foam: (a) before gel foam entered the fracture (WX), (b) gel foam entered the fracture (WX), (c) the fracture (WX) plugged by gel foam.

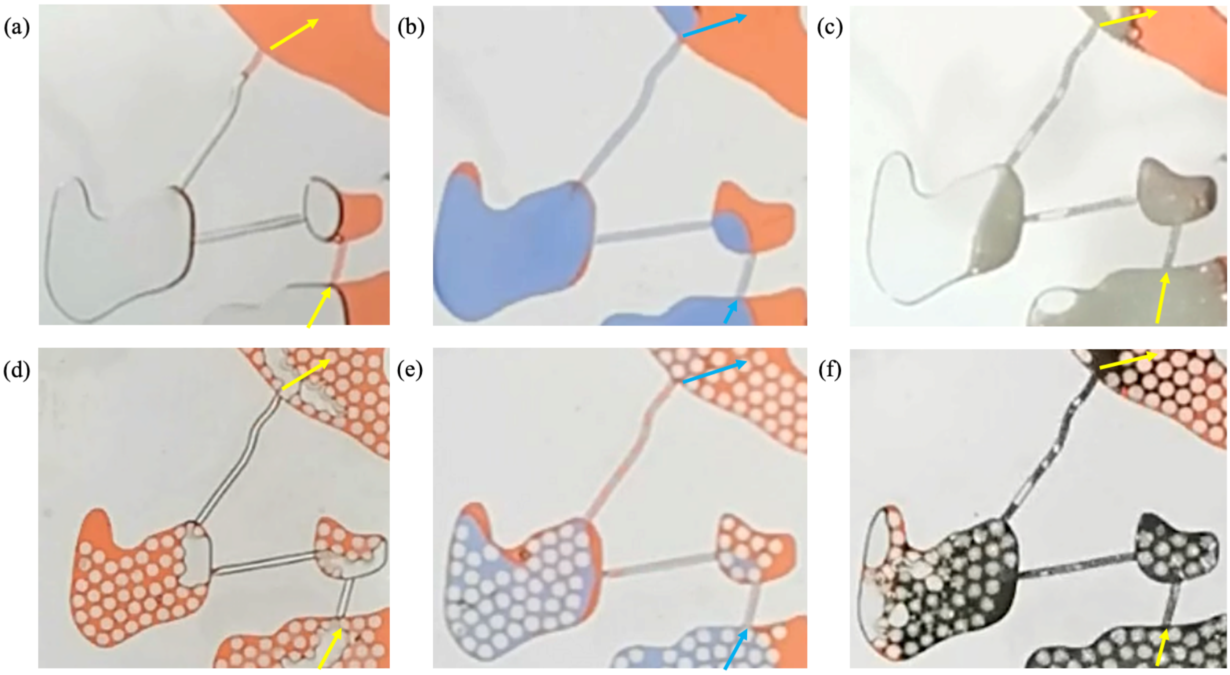

Figure S10. The flow behavior of different fluid in fractured-vuggy structure: (a) gas flowed in unfilled model, (b) water flowed in unfilled model, (c) gel foam flowed in unfilled model, (d) gas flowed in filled model, (e) water flowed in filled model, (f) gel foamed flow in filled model.

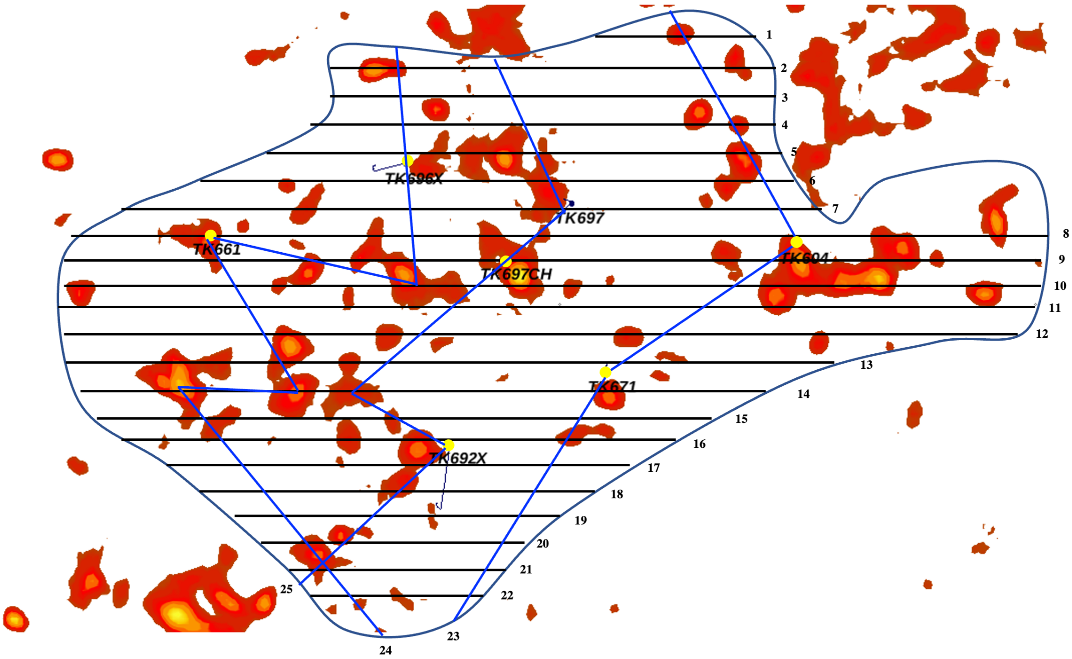

Figure S11. Schematic diagram of the sectional position of the TK671 well-group unit.

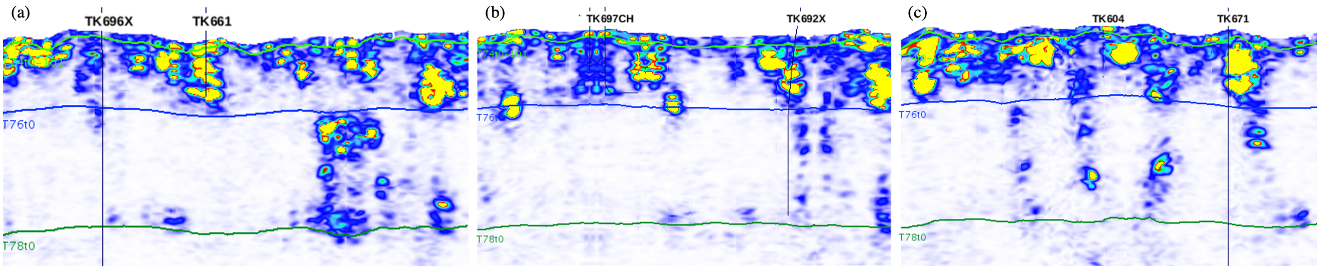

Figure S12. The over-well sections of the TK671 well-group unit: (a) line.23, (b) line.24, (c) line.25.

2. Supplementary table

Table S1. The experimental values of water flooding and gas flooding in fractures

Experimental methods	Contact angle (θ_1, °)	Capillary radius (r_1, m)	Interfacial tension (σ, mN/m)	Capillary force (P_c, Pa)
Testing method	/	/	Spinning drop method; Hanging plate method	/
Gas flooding	18	1.5	27.49	34.86
Water flooding	58	2.6	21.75	8.87
